# *GRIN2A* and Schizophrenia: Scientific Evidence and Biological Mechanisms

**DOI:** 10.2174/011570159X327712241023084944

**Published:** 2024-11-04

**Authors:** Xiao-Ming Sheng, Wei Guan

**Affiliations:** 1 Department of Trauma Center, Affiliated Hospital of Nantong University, Nantong 226001, Jiangsu, China;; 2 Department of Pharmacology, Pharmacy College, Nantong University, Nantong 226001, Jiangsu, China

**Keywords:** *GRIN2A*, Schizophrenia, NMDAR, pathophysiology, synaptic signalling, mutations

## Abstract

Schizophrenia is a severe psychiatric disorder and a complex polygenic inherited disease that affects nearly 1% of the global population. Although considerable progress has been made over the past 10 years in the treatment of schizophrenia, antipsychotics are not universally effective and may have serious side effects. The hypofunction of glutamate NMDA receptors (NMDARs) in GABAergic interneurons has long been postulated to be the principal pathophysiology of schizophrenia. A recent study has shown that *GRIN2A* pathogenic variants are closely related to the aetiology of the disorder. *GRIN2A* encodes the GluN2A protein, which is a subunit of NMDAR. Most *GRIN2A* variants have been predicted to cause protein truncation, which results in reduced gene expression. Preclinical studies have indicated that *GRIN2A* mutations lead to NMDAR loss of function and substantially increase the risk of schizophrenia; however, their role in schizophrenia is not well understood. We hypothesise that the heterozygous loss of *GRIN2A* induces NMDAR hypofunction sufficient to confer a substantial risk of schizophrenia. Therefore, this review focuses on *GRIN2A* as a target for novel antipsychotics and discusses the mechanisms by which *GRIN2A* modulates antischizophrenic activities. Moreover, our review contributes to the understanding of the pathophysiology of schizophrenia to facilitate finding treatments for the cognitive and negative symptoms of schizophrenia.

## 1 INTRODUCTION

Schizophrenia is one of the most serious mental disorders with a high morbidity rate that detrimentally affects a significant portion of the worldwide population [1]. The onset of schizophrenia typically occurs in adolescence or early adulthood and is mainly caused by both genetic and environmental stimuli [2]. Schizophrenia is a complex neuropsychiatric disorder characterized by wide-ranging positive, negative, and cognitive symptoms [3, 4]. Positive symptoms include hallucinations, delusions, and paranoia, while negative symptoms include loss of pleasure, impaired language production, and abnormalities in social interaction [5]. Negative symptoms often appear earlier than any other symptom and are a cardinal, enduring, and debilitating component of the psychopathology of schizophrenia [6]. However, recent studies have shown that cognitive impairment is one of the core symptoms of schizophrenia because almost all (98%) people with schizophrenia (PWS) showed cognitive decrement compared to their premorbid state [7, 8]. Alterations in multiple cognitive domains occur throughout the illness, pre-dating psychosis onset and sustaining the illness even after symptomatic remission [9].

Schizophrenia is a neurodevelopmental disorder thought to be associated with abnormal neurophysiology, including increased presynaptic dopamine synthesis [10], glutamatergic neurotransmission-induced synaptic dysfunction [11], aberrant serotonin signalling [12], gamma-aminobutyric acid (GABA) signalling disturbances [13], inflammation and oxidative stress [14]. Considerable progress has been made in the treatment of schizophrenia over the past 10 years, including psychosocial treatment, effective pharmacologic treatment, and electroconvulsive therapy [15, 16]. In addition, adjunct music therapy and dance/movement therapy have emerged as an important therapeutic intervention to improve the quality of life of patients with schizophrenia [17, 18]. It is universally accepted that the interactions of antipsychotics with various neurotransmitter receptors are responsible for their effects on the treatment of schizophrenia symptoms [19]. Although antipsychotics are the first-line treatment for schizophrenia (mainly for ameliorating positive symptoms) [20], these classes of drugs are not universally effective against negative and cognitive symptoms. In addition, they have a high risk of metabolic side effects, such as diabetes, hypercholesterolaemia, and weight gain, and may lead to neurologic side effects (tardive dyskinesia) [21, 22]. Therefore, new therapeutic agents that reduce negative symptoms and increase the quality of life of patients are urgently needed.

*GRIN2A*, which encodes GluN2A (an important subunit of NMDARs), plays an important role in schizophrenia [23]. NMDARs are di- or triheterotetrameric ligand-gated ion channels composed of two obligate GluN1 subunits (encoded by *GRIN1*) and two glutamate-binding GluN2 subunits (encoded by *GRIN2A*-*D)*, respectively [24]. Accumulating evidence indicates that NMDAR is the main receptor of the excitatory neurotransmitter glutamate in the brain and is a key determinant of the excitatory/inhibitory (E/I) balance of neural networks [25, 26], whereas NMDAR dysfunction and E/I imbalance are thought to be the major pathophysiological mechanisms of schizophrenia in pharmacologic and genome-wide association studies [27]. Trubetskoy *et al*. performed the largest Genome-Wide Association Study (GWAS) of schizophrenia to date and, in doing so, identified *GRIN2A* as a specific gene where the convergence of rare and common variant associations strongly supports their pathogenic roles in the pathogenesis of schizophrenia [28]. Additionally, sequencing data have shown that 43% of all currently known NMDAR disease-associated genetic variants are within *GRIN2A* [29], further indicating that *GRIN2A* is one of the most highly prioritised genes related to the pathophysiology of schizophrenia [30]. Furthermore, recent studies by Poltavskaya *et al*. showed that *GRIN2A* rs11644461 and rs8057394 are associated with the continuous type of schizophrenia [31], and *GRIN2A* rs7206256 is linked to the early manifestation of this disease [32]. However, a more precise understanding of the pathomechanisms of schizophrenia is lacking. Therefore, in this review, we discuss the relationship between mechanisms common to schizophrenia and *GRIN2A* function. In summary, this review aims to develop a better understanding of the mechanisms underlying schizophrenia and identify promising targets that may facilitate the development of new treatments.

## 2 NMDAR STRUCTURE AND FUNCTION

## 2.1 Structure

**Fig. (1):**
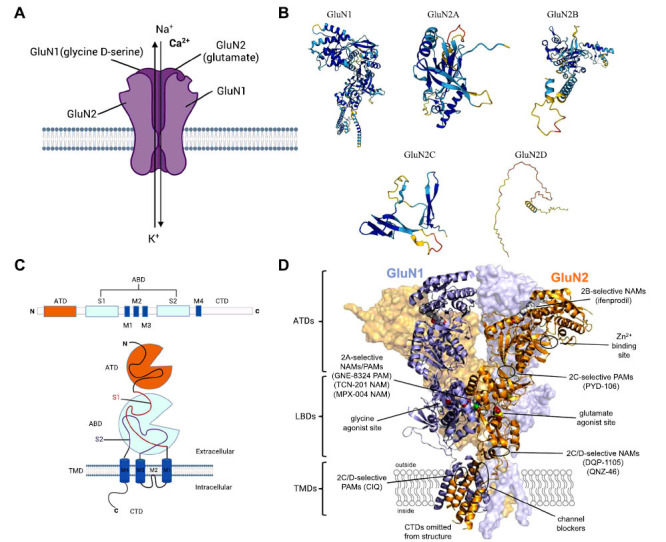
Subunit composition of GluN1/2 NMDA receptors. (**A**) NMDARs are large heterotetrameric protein complexes comprised of different subunits that form a central cation-permeable channel pore, including two obligate GluN1 subunits encoded by *GRIN1* and two GluN2 subunits (GluN2A-GluN2D) encoded by *GRIN2A*-*GRIN2D*. (**B**) The crystal structure of the GluN1 and GluN2 subunits (created with AlphaFold Monomer v2.0). (**C**) All NMDAR subunits share a similar architecture that consist of four domains: a highly homologous extracellular amino-terminal domain (ATD), a bilobed ligand-binding domain (LBD), a pore-forming transmembrane domain (TMD) comprised of three transmembrane regions and a re-entrant ion pore-lining loop, and an intracellular carboxyl-terminal domain (CTD). (**D**) The crystal structure of the GluN1/GluN2 NMDAR (Protein Data Bank accession no. 4PE5; [102]) shows the subunit arrangement and the layered domain organization. Agonist binding sites as well as known and predicted binding sites for positive and negative allosteric modulators (PAMs and NAMs) are highlighted. Panels (**C**) and (**D**) are adapted from Hansen *et al*. [103]. The figure was generated using BioRender (Agreement number: HG27790VKO).

NMDARs are glutamate-gated ion channels widely expressed in the Central Nervous System (CNS) that play key roles in excitatory synaptic transmission. NMDARs are large heterotetrameric protein complexes comprising different subunits that form a central cation-permeable channel pore, including two obligate GluN1 subunits encoded by *GRIN1* and two GluN2 subunits (GluN2A-GluN2D) encoded by *GRIN2A*-*GRIN2D* (Figs. **1A**, **B**) [33]. Additionally, they may include GluN3 subunits encoded by *GRIN3A* and *GRIN3B* [34]. Some studies have identified two types of GluN3-containing NMDARs: glutamate-gated GluN1/GluN2/ GluN3 and glycine-gated GluN1/GluN3 NMDARs [23]. All NMDAR subunits share a similar architecture consisting of four domains: a highly homologous extracellular Amino-Terminal Domain (ATD), a bilobed Ligand-Binding Domain (LBD), a pore-forming Transmembrane Domain (TMD) comprising three transmembrane regions, a re-entrant ion pore-lining loop, and an intracellular carboxyl-terminal domain (Figs. **1C** and **1D**) [35]. Due to the lower understanding of GluN3-containing NMDARs compared to those composed of GluN1 and GluN2 subunits, GluN3 subunits will not be discussed in this review. Given that GluN1 subunits are widely expressed in virtually all central neurons, they are obligatory subunits of all functional NMDARs [36]. The GluN2 subunit contains a glutamate-binding site and controls the pharmacological and biophysical properties of NMDARs [35]. Interestingly, GluN2 subunits (GluN2A-GluN2D) display striking differences in their spatiotemporal expression patterns in the brain, as well as unique pharmacologic and functional properties [37, 38]. These differences determine the time course of NMDAR-mediated excitatory postsynaptic currents, which affect synaptic integration and plasticity in various brain regions [35].

## 2.2 Function

NMDARs are a subclass of ionotropic glutamate receptors (iGluRs) that respond to the neurotransmitter glutamate and are permeable to sodium (Na^+^), potassium (K^+^), and calcium (Ca^2+^) ions [35]. Research has shown that they play a crucial role in calcium ion permeability and excitatory neurotransmission in the CNS [39]. Upon heightened neuronal activity, the blockade of Mg^2+^ is relieved, leading to the opening of NMDARs, which mediates the flux of Ca^2+^ into the postsynaptic compartment [40]. Subsequently, Ca^2+^ influx serves as a signal that underpins multiple forms of synaptic plasticity [41]. NMDAR activation requires the simultaneous binding of glycine/D-serine and glutamate to the GluN1 and GluN2 subunits, respectively [35]. Compared with GluN1, the GluN2 subtype regulates a wide variety of NMDAR biophysical properties, and these distinctions underpin synaptic plasticity and integration in various regions of the brain.

**Fig. (2):**
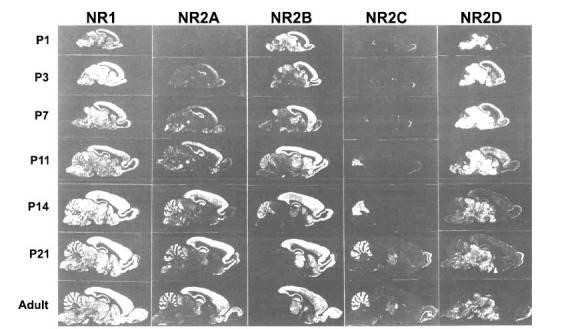
Autoradiograms of *in situ* hybridization conducted with oligonucleotide probes on parasagittal sections of rat brains on designated postnatal (P) days (day 1, 3, 7, 11, 14, 21, and 56). The figure shows the distributions of mRNAs for the five NMDA receptor subunits: GluN1 (NR1), GluN2A (NR2A), GluN2B (NR2B), GluN2C (NR2C), and GluN2D (NR2D). GluN2A is predominantly expressed in the forebrain at birth and progressively increases throughout the CNS until adulthood. While the expression of GluN2B is widely distributed at an early stage and reaches its peak at approximately P7. By comparison, GluN2C expression is confined to the cerebellum and the olfactory bulb and starts in the second postnatal week [44]. GluN2D expression is found mostly in the mid-brain structures such as the diencephalon and mesencephalon, while it is significantly decreased in the adult. The figure is adapted from Akazawa *et al*. [44]. Scale bar = 10 mm.

Interestingly, GluN2 subunits display developmentally and spatially regulated expression patterns in different brain regions (Fig. **2**) [37]. Research has shown that GluN2A is predominantly expressed in the forebrain at birth, and its expression progressively increases throughout the CNS until adulthood, whereas the expression of GluN2B is widely distributed at an early stage and peaks around postnatal day 7 (P7) [42]. Additionally, the expression of GluN2B is restricted to the forebrain during adulthood. Therefore, NMDARs are believed to have evolved from being predominantly GluN2B-containing to GluN2A-containing during development in the hippocampus or cortex [43]. In contrast, GluN2C expression is confined to the cerebellum and olfactory bulb and begins in the second postnatal week [44]. GluN2D expression is found mostly in midbrain structures, such as the diencephalon and mesencephalon, whereas it is significantly decreased in adults [44]. Among the GluN2 subunits, much attention has been focused on GluN2A.

The *GRIN2A* gene encodes the GluN2A protein, and Loss-of-Function (LoF) mutations in *GRIN2A* are associated with a spectrum of psychiatric disorders, including schizophrenia [45]. In this review, we highlight recent studies on the role of *GRIN2A* in the pathogenesis of schizophrenia. This knowledge is vital for understanding the extraordinarily complex mechanisms of schizophrenia to develop neuroprotective drugs with optimal therapeutic profiles.

## 3 *GRIN2A* AND SCHIZOPHRENIA

**Fig. (3):**
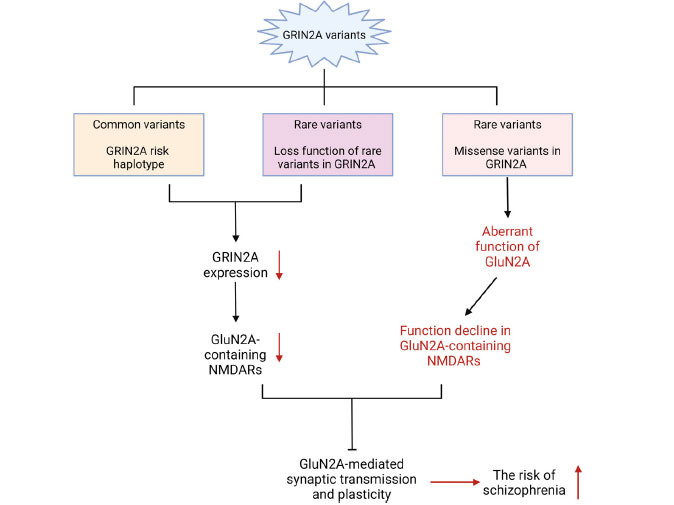
The schematic diagram for the functional consequences of *GRIN2A* variants involved in schizophrenia. *GRIN2A* variants, including common and rare variants, were proposed to lead to reduced *GRIN2A* expression and GluN2A-containing NMDARs, while the missense variants in *GRIN2A* would cause aberrant GluN2A function and thence impaired GluN2A-containing NMDARs. Finally, these function declines in GluN2A-containing NMDARs might reduce the synaptic transmission and plasticity, and consequently result in the onset of schizophrenia.

Previous studies have confirmed that *GRIN2A* is predominantly associated with neurodevelopmental disorders, including epilepsy and intellectual disability [46]. However, strong evidence provided by recent studies has implicated rare pathogenic deletions, including those in *GRIN2A*, in adult-onset schizophrenia [46, 47]. Rare heterozygous LoF mutations in *GRIN2A*, which encodes a subunit of the NMDAR, significantly increase the risk of schizophrenia (Fig. **3**). Although previous research has revealed a relationship between *GRIN2A* pathogenic variants and schizophrenia in some cases, the underlying mechanism remains unknown. Therefore, we focused on the underlying biological mechanisms of *GRIN2A* variants in schizophrenia in the upcoming chapter.

### 3.1 *GRIN2A* Variants in Patients with Early Onset of Schizophrenia

Schizophrenia is a complex neurodevelopmental condition that usually occurs in late adolescence and early adulthood [2]. However, a recent study showed that symptoms also occur in childhood and adolescence before the ages of 13 and 18 years, respectively [47]; this condition is commonly known as early-onset and childhood-onset schizophrenia. In 2023, Hojlo *et al*. published the first study of rare *GRIN2A* variants in children and adolescents with Early-Onset Psychosis (EOP). Whole exome sequencing of DNA from peripheral blood samples revealed that *GRIN2A* variants were significantly increased in the EOP cohort [47]. These findings indicate that *GRIN2A* variants are associated with childhood-onset schizophrenia. However, the researchers also noted that the *GRIN2A* variants in EOP were not pathogenic, except for one variant of unknown significance (chr16-9934641 G>T, p.Ala505Glu), according to the American College of Medical Genetics (ACMG) criteria. As these guidelines were not developed for the interpretation of variants in genes associated with complex disorders, a larger cohort should be studied. Consistent with the above results, Poltavskaya *et al*. performed genetic analysis of blood samples after 8 h of overnight fasting and showed that the rs7206256 and rs11644461 polymorphisms of the *GRIN2A* gene were associated with early onset of schizophrenia (before the age of 18 years) [32]. However, in their study, *GRIN2A* polymorphisms were not related to predominant positive or negative symptoms or the course of schizophrenia.

In contrast, Poltavskaya *et al*. indicated that *GRIN2A* rs11644461 and rs8057394 were associated with the continuous type of schizophrenia (ages 18-60) [31]. Further research showed that the *GRIN2A* rs8057394*G allele was a relative risk factor for the development of the continuous type of schizophrenia (*p* = 0.019), whereas the *GRIN2A* rs11644461*T allele was shown to have a protective effect against the development of the continuous type of schizophrenia. Finally, the rs9788936 and rs8057394 polymorphisms of *GRIN2A* were found to be associated with the intensity of symptoms in schizophrenia, based on Positive and Negative Syndrome Scale (PANSS) scores [31]. These findings indicate that individual *GRIN2A* variations are related to the development of clinical manifestations of schizophrenia.

### 3.2 Common EEG Phenotypes in *GRIN2A* Mutant Mice and Schizophrenia Patients

**Fig. (4):**
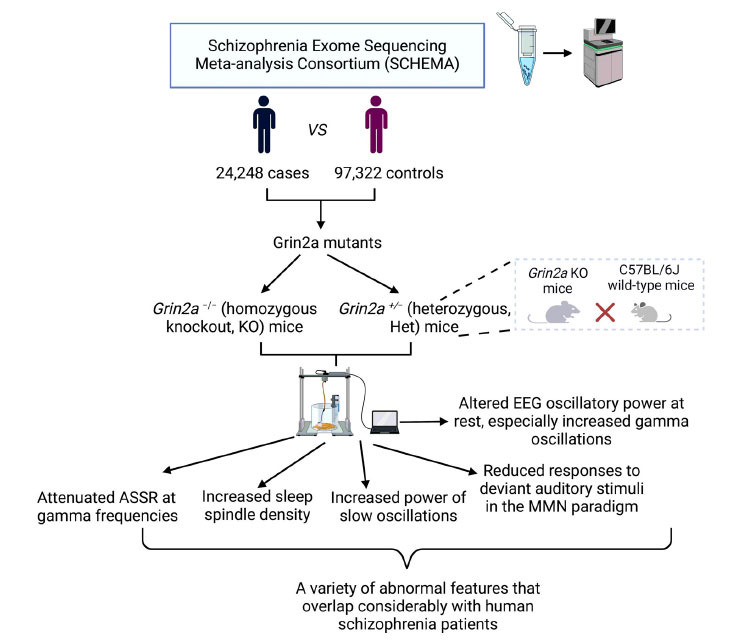
The Schizophrenia Exome Sequencing Meta-Analysis Consortium (SCHEMA) has identified multiple rare loss-of-function genetic variants in one of the largest sequencing studies to date of 24,248 cases and 97,322 controls, such as *GRIN2A* mutations [30]. Herzog *et al*. found *GRIN2a^+/^*^−^ and ^−^*^/^*^−^ mice exhibited gene dose-dependent increases in gamma oscillations and sleep spindles compared with WT [45]. Moreover, relative to WT, *GRIN2a* mutants showed attenuated auditory steady-state responses (ASSR) at gamma frequencies and reduced responses to deviant auditory stimuli in the Mismatch negativity (MMN) paradigm [45]. The EEG phenotypes of *GRIN2a* mutant mice showed a variety of abnormal features that overlapped considerably with human schizophrenia patients. The figure was generated using BioRender (Agreement number: KV27791A2V).

Electroencephalography (EEG) is a low-resolution diagnosis tool and is especially useful for understanding abnormalities in brain disorders, especially schizophrenia [48]. A recent study revealed that *GRIN2A* mutant mice showed a variety of abnormal EEG phenotypes that were also observed in patients with schizophrenia (Fig. **4**) [45]. In the study, *Grin2a^−/−^* (homozygous knockout, KO) and *Grin2a^+/−^* (heterozygous) mice and their wild-type (*Grin2a* WT) littermates were used to conduct the open field test and EEG recording. Researchers found *Grin2a* KO mice showed increased locomotor activity in the open field test, whereas they had reduced center/margin distance ratios compared to their WT littermates. However, there was no significant difference in the total distance travelled and the ratio of center/margin distance between the *Grin2a^+/−^* mice and their WT littermates [45]. Further experiments revealed that *Grin2a^−/−^* mice showed a significant increase in resting gamma power compared with their WT littermates [45], which is frequently observed in many patients with schizophrenia at rest [49]. In addition, they also found that the sleep spindle density in *Grin2a* mutants significantly increased in a gene dose-dependent manner. *Grin2a* mutant mice exhibited attenuated auditory steady-state responses at gamma frequencies and showed reduced responses to deviant auditory stimuli in the Mismatch Negativity (MMN) paradigm compared with *Grin2a^+/−^* and WT mice [45]. Previous studies have consistently supported a link between MMN and psychosocial functioning in schizophrenia [50]. As a biomarker for schizophrenia, MMN is significantly smaller in individuals with chronic schizophrenia than in healthy controls [51]. These findings indicate that *Grin2a* KO mice show EEG abnormalities and that EEG recordings serve as biomarkers of schizophrenia.

Consistent with the notion that *Grin2a* KO mice showed EEG abnormalities, Salmi *et al*. showed an increased occurrence of High-Voltage Spindles (HVSs) in *Grin2a* KO mice in the 3rd postnatal week compared to WT mice in-depth EEG intracortical recordings [52]. Moreover, *Grin2a* KO mice spent significantly less time in slow-wave sleep than WT mice on P30, whereas this reduction was no longer observed in the same mice at P60 [52]. Accumulating evidence indicates that sleep spindle deficits are correlated with impaired sleep-dependent memory consolidation and positive symptoms in schizophrenia [53]. In addition, slow-wave abnormalities are commonly observed in patients with schizophrenia, especially when they experience an exacerbation of psychosis [54]. Generally, pathogenic variants of *GRIN2A* lead to NMDAR dysfunction, which is associated with schizophrenia onset.

### 3.3 The Function of *GRIN2A* in Neuroinflammation

Several studies have shown that CNS inflammation may play a role in the pathogenesis of schizophrenia [55]. Inflammatory markers such as proinflammatory cytokines are well-known etiological factors of schizophrenia [56]. During the inflammatory response, some proinflammatory cytokines alter neurotransmitter metabolism or increase the production of neurotoxic pathways across the Blood-Brain Barrier (BBB) [57]. For example, a previous study revealedthat overexpression of interleukin 6 (IL-6) inhibits hippocampal neurogenesis [58], whereas impaired neurogenesis may indicate a deteriorating course of schizophrenia [59]. Moreover, recent studies have shown that neuroinflammation is invariably linked to the activation of tryptophan metabolism through the kynurenine (KYN) pathway, whereas disturbances in KYN metabolites have been linked to psychiatric disorders [60]. Current evidence suggests that increased kynurenic acid levels are found in the brains of patients with schizophrenia [61], which consequently leads to antagonism of NMDA receptors and a lack of glutamate neurotransmission [62]. However, the mechanisms underlying neuroinflammation in schizophrenia remain unknown, and more comprehensive and in-depth studies are required.

**Fig. (5):**
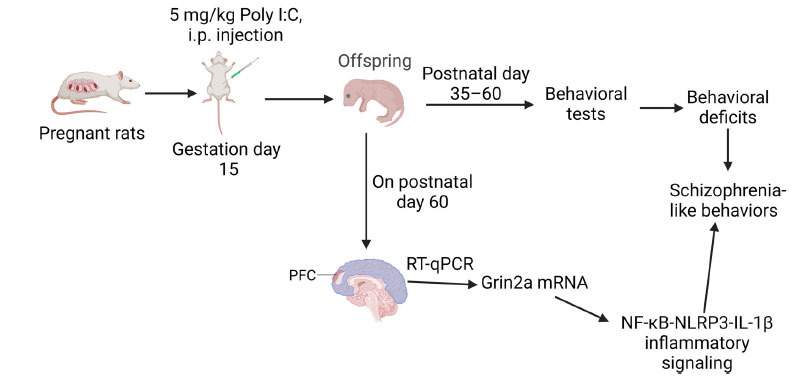
Pregnant rats exposed to polyriboinosinic-polyribocytidylic acid (Poly I:C; 5 mg/kg) at gestation day (GD)15 showed schizophrenia-like behaviors and abnormal neurotransmissions (increased mRNA expression of the *Grin2a* subunit) in the prefrontal cortex (PFC) *via* elevated neuroinflammation responses (NF-κB-NLRP3-IL-1β inflammatory signaling) [63]. **Abbreviations:** NF-κB: nuclear factor kappaB; NLRP3: pyrin domain containing 3; qRT-PCR: quantitative real-time PCR. The figure was generated using BioRender (Agreement number: NX27791J5S).

According to a previous study, pregnant rats exposed to polyriboinosinic-polyribocytidylic acid (Poly I:C; 5 mg/kg) on gestational day (GD) 15 exhibited schizophrenia-like behaviours and abnormal neurotransmission (increased mRNA expression of the *Grin2a* subunit) in the prefrontal cortex (PFC) *via* elevated neuroinflammation responses (Fig. **5**) [63]. Interestingly, schizophrenia-related behaviour and neurotransmitter receptor expression in offspring following prenatal Poly I:C exposure appears to depend on the administration time and dosage. For instance, Poly I:C treatment (4.0 mg/kg) on GD9/10 caused dopamine-related positive symptom-like behavioural changes and increased dopamine 1 receptor mRNA levels in the nucleus accumbens of male rats [64], whereas exposure during late gestation (*e.g*., GD15, GD17, and GD19) caused phenotypes in the offspring that were more consistent with negative/cognitive symptoms of schizophrenia and NMDAR alterations [64, 65]. On the other hand, Gogos *et al*. [66] revealed that 4 mg/kg of Poly I:C injected into the tail vein at a volume of 1 mL/kg on GD15 caused increased psychosis-like behaviour in maternal immune activation (MIA)-induced rats. Poly I:C is delivered *via* intraperitoneal injection in most studies using the MIA rat model [63, 67]. This indicated there was no significant difference in offspring phenotypes in models generated *via* the two Poly I:C administration routes (intraperitoneal and intravenous). Consistent with this, Liang *et al*. [68] found that Interleukin-1 Receptor-Associated Kinase (IRAK)-M deficiency promoted a microglia polarization shift toward the M1 phenotype. This led to the release of proinflammatory cytokines and was accompanied by a visible increase in glutamatergic synaptic transmission (GluN2A) in the hippocampal CA1 of the *IRAK-M^−/−^* mice. Recent studies have demonstrated that IRAK-M is an important negative regulator of innate immunity and is closely associated with certain neurological disorders [69]. These results suggested that higher levels of proinflammatory cytokines increase the function of NMDARs in postsynaptic neurons, leading to excitotoxicity [70].

In contrast, some studies have shown that NMDAR hypofunction, such as decreased expression of *GRIN2A*, aggravated the symptoms of schizophrenia [71]. Shishkina *et al*. [72] determined that transient lipopolysaccharide (LPS) treatment (30 µg for 24 h) induced memory impairment and a significant elevation in the expression of the proinflammatory cytokine interleukin-1 beta (IL-1β) in the right striatum and hippocampus of Wistar rats, revealing neuroinflammation is involved in the dysregulation of neurogenesis through distinct neurobiological mechanisms [73]. Furthermore, they discovered that pretreatment with dexamethasone (DEX; 5 mg/kg intraperitoneally; 30 min before the administration of LPS) significantly increased *Grin2a* expression and improved memory in DEX-pretreated rats [72]. Indeed, GluN2A-containing hippocampal NMDARs have been reported to play important roles in synaptic plasticity and learning and memory, as evidenced by previous research [74]. However, it is worth noting that there was no significant difference in the expression of *Grin2a* in rats that received LPS or DEX alone compared with control animals, suggesting that pretreatment with DEX did not markedly affect the LPS-induced prolonged inflammatory response, which was likely due to the poor penetration of DEX into the brain [75]. In contrast, *Grin2a* expression was significantly elevated in the DEX + LPS group compared to that in the LPS group, possibly because the LPS-induced increase in BBB permeability enhanced brain susceptibility to DEX. These results suggest that DEX pretreatment activates regulators of glutamate, such as GluN2a, which is possibly involved in the recovery of memory impairment induced by LPS. Moreover, another preclinical study showed that the ratio of *GRIN2A: GRIN2B* mRNA ratio was decreased in postmortem dorsolateral PFC sections of patients with schizophrenia, and this reduction was most apparent in schizophrenia cases with a high inflammatory status [71]. This suggests that a high GluN2A:GluN2B ratio is associated with neuroprotection [76]. These inconsistent findings may be associated with methodological differences, sample sizes, or sample types. Further investigations are necessary to elucidate the underlying molecular mechanisms.

### 3.4 The Function of *GRIN2A* in the Maturation of Parvalbumin (PV) Interneurons (PVIs)

Existing studies have shown that mutations and polymorphisms in *GRIN2A*, coding for GluN2A, are closely associated with the pathogenesis of schizophrenia [32, 77]. Moreover, there are connections between GluN2A and PVIs, as supported by several recent lines of evidence [78]. GluN2A is involved in the maturation and phenotypic maintenance of PVIs, whereas dysfunction of glutamatergic GluN2A-containing NMDARs during neurodevelopment in schizophrenia can disrupt the maturation of these interneurons [78]. PVIs are fast-spiking, nonadapting interneurons that express PV (a high-affinity calcium-binding protein) [79]. Because they form inhibitory synapses with either the cell body or the axonal initial segment of pyramidal neurons, PVIs exert precise temporal control over information flowing through their target neurons *via* feed-forward and feedback inhibition [78, 79]. It has been demonstrated that PVI activity synchronises with the medial PFC circuit to facilitate normal cognitive function [80], whereas aberrant activity is a key pathological feature of schizophrenia. This indicates that anomalies in PVIs are a pathological hallmark of schizophrenia. In support of this, alterations in PVIs were highly replicated in the postmortem brains of patients with schizophrenia [81, 82]. Although published data support the role of GluN2A in the development and phenotyping of PVIs, the precise mechanisms underlying the regulation of the molecular networks relevant to schizophrenia are unknown.

**Fig. (6):**
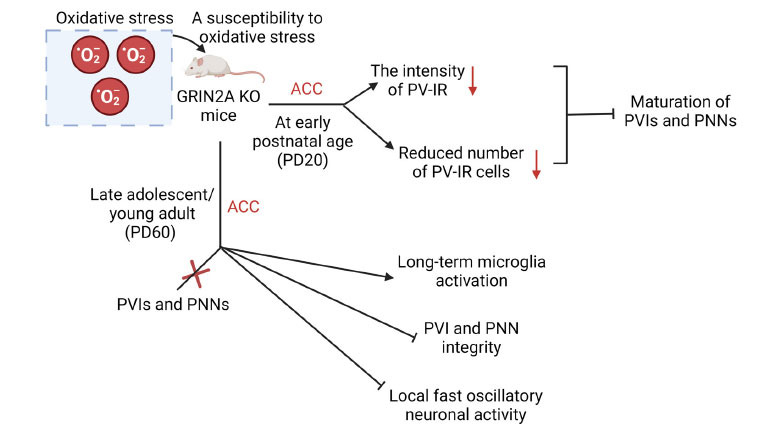
It was found that a lack of GluN2A delayed the maturation of PVIs and PNNs in *GRIN2A* KO mice, while PVIs and PNNs appeared normal in ACC of late adolescent/young adult *GRIN2A* KO mice [78]. Moreover, *GRIN2A* KO mice at early postnatal age (PD20) showed weakened antioxidant defenses and greater vulnerability to an early postnatal oxidative insult as compared to WT mice. Finally, an early postnatal oxidative insult had long-term effects on PVI and PNN integrity, microglia activation, and local fast oscillatory neuronal activity in *GRIN2A* KO mice at late adolescence/early adulthood (PD60) [78]. **Abbreviations:** PVIs: parvalbumin-expressing interneurons; PNNs: perineuronal nets; ACC: anterior cingulate cortex. The figure was generated using BioRender (Agreement number: HK27791QG9).

The possible connections between GluN2A-containing NMDARs and PVIs have been investigated in recent studies [83]. According to previous studies, GluN2A-containing NMDARs contribute to PVI maturation and phenotypic maintenance [78, 84], while the absence of GluN2A causes susceptibility to oxidative stress and consequently leads to the behavioural phenotypes of schizophrenia. Notably, oxidative stress is a common pathological mechanism that leads to PVI impairment in schizophrenia [85]. Specifically, research conducted by Cardis *et al*. [78] demonstrated that *Grin2A* KO mice displayed susceptibility to redox dysregulation, and an early life oxidative insult leads to persistent oxidative stress together with long-term effects on the PVIs and Perineuronal Net (PNN) integrity in the Anterior Cingulate Cortex (ACC) of *Grin2A* KO mice (Fig. **6**). Furthermore, the authors also found that the absence of GluN2A delayed the maturation of PV-immunoreactive (PV-IR) neurons and PNNs in the ACC of young *Grin2A* KO mice (P20) as compared with WT mice at P70, suggesting that an early life oxidative insult might lead to delayed maturation of PNNs, which have been demonstrated to protect PVIs against oxidative stress [86]. These results indicate that GluN2A favours PNN and PVI maturation, which is consistent with the notion that NMDAR subunits contribute significantly to the function of GABAergic interneurons, particularly PVIs. In addition to genetic and environmental factors, early life stressors may play a role in the severity of schizophrenia pathology. However, it should be noted that lack of GluN2A had no impact on PV-IR and PNNs in late adolescence or early adulthood, highlighting the fact that GluN2A expression increases after birth in an activity-dependent manner, and this subunit becomes dominant in adulthood [87]. Further investigations are warranted to validate these findings and explore the potential of GluN2A as a diagnostic or therapeutic target in schizophrenia.

This result was confirmed by Zhang *et al*. [83]. They discovered that chronic blockage of NMDAR receptor subunit NR2A (with NVP-AAM077, 1.2 mg/kg delivered intraperitoneally), but not NR2B, in GAD67-GFP-positive mice aged P7 to P25 reduced the density of PV-positive interneurons in layer 2/3 and layer 4 barrel cortex [83], demonstrating that NR2A-containing NMDARs play a pivotal role in the maturation of PV-positive cells *in vivo*. Taken together, these results indicate a role for GluN2A, which is enriched in PVIs, in the regulation of molecular networks relevant to schizophrenia.

### 3.5 The Function of *GRIN2A* in Maintaining Synaptic Levels of NMDARs

According to previous studies, synaptic pathology is a feature of the brain in patients with schizophrenia that manifests as alterations in the expression of synaptic proteins [88]. Recent studies have implicated variations in genes encoding synaptic pathways in the pathogenesis of schizophrenia [89]. NMDARs play important roles in synaptic plasticity under both physiological and pathological conditions. GluN2A, the most expressed NMDAR regulatory subunit in the hippocampus, plays a pivotal role in excitatory synaptic transmission and plasticity in the mammalian CNS [90], and its rare variants greatly increase the risk of schizophrenia.

According to one study, mRNA levels of *Grin2a* in the hippocampus of inbred Roman high- (RHA-I) rats were lower than those of Roman Low- (RLA-I) rats [91]. The RHA-I rat strain is a behavioural model that presents schizophrenia-like traits [92]. Furthermore, the expression of presynaptic markers such as Vamp1 and Snapin was significantly downregulated in the hippocampus of RHA-I rats compared with RLA-I rats, while there was no significant difference in the expression of postsynaptic markers such as Psd95 and Nrg1 [92]. However, brain-derived neurotrophic factor (BDNF) protein levels were significantly increased in the hippocampus but decreased in the PFC of RHA-I rats compared with RLA-I rats. BDNF is a neuropeptide that plays an important role in synaptic development and plasticity [93]. Regarding this discrepancy, the authors speculated that RHA-I rats showed increased BDNF transcription in the hippocampus to compensate for the altered posttranscriptional regulation of BDNF in the PFC.

Additionally, a study conducted on hippocampal neuronal cultures from pregnant rats demonstrated that reduced GluN2A expression delays the maturation of hippocampal neurons [87]. Further analysis indicated that GluN2A knockdown (AAV-sh2A) induced an increase in the number of immature spines but reduced synaptic NMDARs. Finally, increased dendritic complexity was observed in GluN2A knockdown cultures compared with control neurons, while no significant differences were found in the total neuronal area between treatment conditions [87]. Surprisingly, the distribution of surface-expressed GluN2A (sGluN2A) remained similar in GluN2A knockdown and control cultures, and there was no difference in the ratio of sGluN2A/total GluN2A between the GluN2A knockdown and control cultures [87]. In light of this finding, we speculate that neurons distribute GluN2A subunits at synapses to sustain stable and mature synaptic connections, and further studies should be performed to confirm this hypothesis. Taken together, these results suggest that increased levels of GluN2A are essential for neuronal maturation and induce an increase in dendritic complexity and immature spines.

NMDARs are heterotetramers found in postsynaptic neurons that mediate fast excitatory transmission and synaptic plasticity [94], whereas overactivation of NMDARs located outside the synapse triggers NMDA-mediated Ca^2+^ influx, excitotoxicity, and impaired synaptic plasticity [95], which ultimately results in schizophrenia-like symptoms.

Li *et al*. reported that enhancing GluN2A-type NMDARs impairs long-term synaptic plasticity [96], and GluN2A_ K879R (a rare GluN2A variant) knock-in mice exhibit defects in synaptic transmission (enhanced excitatory postsynaptic currents). Furthermore, the Lundbye laboratory showed impaired synaptic plasticity in the *Fmr‐1 knock‐out* (*Fmr1-/y*) mouse was restored by pharmacologically (using TCN‐201 or NVP‐AAM077) inhibiting GluN2A‐containing NMDARs [97], and similar results were observed by cross‐breeding *Fmr1-/y* with *GRIN2A-/-* mice. These results indicate that dampening the elevated levels of GluN2A‐containing NMDARs might restore the hyperexcitability of the neural circuitry to normal levels of brain activity.

## CONCLUSION

In conclusion, existing research emphasizes the significant impact of NMDARs on the pathophysiology of schizophrenia. Numerous genetic and postmortem studies have linked NMDAR dysfunction to schizophrenia, forming the basis for the glutamate hypothesis [98]. Rare and common variants of *GRIN2A*, which encodes the GluN2A subunit of the NMDAR, are associated with genetic risk for schizophrenia. Despite enormous efforts employing various approaches, the molecular pathology of schizophrenia remains unclear. Therefore, in this article, we reviewed the evidence that alterations in glutamatergic neurotransmission (*via* NMDARs), especially focusing on the function of *GRIN2A*, maybe a critical causative feature of schizophrenia.

*GRIN2A* variants are closely associated with childhood- or adult-onset schizophrenia [47]. *GRIN2A* variants were significantly increased in the EOP cohort in a sequence kernel association test [47]. Moreover, there is a significant linkage and association between a functional (GT)n polymorphism in the promoter of *GRIN2A* and schizophrenia [99]. This finding was supported by the research from Itokawa *et al*. on *GRIN2A*; they identified a variable (GT)n polymorphism in the 5′-regulatory region of *GRIN2A* [100]. Compared with control subjects, the (GT)n polymorphism in the promoter of *GRIN2A* showed marginally significant allelic differences in patients with schizophrenia, which reduced the expression of *GRIN2A* [100]. However, these effects are not consistently observed, and a recent preclinical study showed that the selected single nucleotide variant in *GRIN2A* encoding a subunit of the NMDAR was not associated with the presence of cognitive deficits in patients with schizophrenia [101]. We speculate that the cognitive impairment observed in these patients may be associated with other genetic conditions or the influence of environmental factors. Other studies have pointed out that there is no connection between *GRIN2A* polymorphisms (rs7206256 and rs11644461) and predominant (positive *vs*. negative) symptoms or type of course (continuous *vs*. episodic) of schizophrenia [32]. Given these findings, we speculate that the dysfunction of GluN2A due to significant mutations in the *GRIN2A* gene might serve as a trigger for the onset of schizophrenia; however, more research is needed to identify the mechanisms in a larger sample.

In summary, in this article, we review studies that demonstrate that rare and common variants of *GRIN2A* are both robustly associated with the genetic risk for schizophrenia. We speculate that the consequence of GluN2A dysfunction induced by mutations in *GRIN2A* is the delayed maturation of PVIs, thereby leading to the impairment of synaptic plasticity, which might contribute to the development of the disease.

## LIST OF ABBREVIATIONS

ATD: Amino-terminal Domain
BBB: Blood-brain Barrier
BDNF: Brain-derived Neurotrophic Factor
CNS: Central Nervous System
EEG: Electroencephalography
EOP: Early-onset Psychosis
GABA: Gamma-aminobutyric Acid
HVSs: High-voltage Spindles
LoF: Loss-of-function
MIA: Maternal Immune Activation
PFC: Prefrontal Cortex
